# Determinants of Premature Rupture of Membranes: A Three-Year Retrospective Analysis of Age, Parity, Delivery Mode, and Seasonality

**DOI:** 10.7759/cureus.85433

**Published:** 2025-06-05

**Authors:** Qin Deng, Guizhen Yu, Lijian Lai, Jiayin Xu, Yuemei Zheng

**Affiliations:** 1 Obstetrics, Dongguan Eighth People's Hospital, Dongguan, CHN; 2 Obstetrics, Dongguan Maternal and Child Health Hospital, Dongguan, CHN

**Keywords:** obstetric care, parturient, premature rupture of membranes (prom), retrospective study, risk factors

## Abstract

Background: Premature rupture of membranes (PROM) precedes 8‑10% of births and remains a leading cause of neonatal morbidity. Although infection, prior obstetric history, and cervical pathology are recognized contributors, data on the relative influence of maternal age, parity, mode of delivery, and seasonality are inconsistent across regions. Clarifying these determinants could enhance antenatal risk stratification and guide preventive counselling.

Methods: We retrospectively reviewed all singleton deliveries recorded in a tertiary care obstetric database from January 2018 through December 2020. Maternal age was grouped as <20, 20‑24, 25‑29, 30‑34, and ≥35 years; parity was dichotomized (primipara vs. multipart). The final mode of delivery (vaginal vs. cesarean) and calendar month of birth were extracted. Incidence of PROM was compared among age groups by the Kruskal-Wallis test, between parity and delivery‑mode strata by χ² tests, and across months by the Friedman test; Holm-Bonferroni corrections addressed multiple comparisons.

Results: Across the three‑year period, PROM complicated 13.7% of deliveries. Incidence varied significantly by age (H = 19.95; p = 0.00051), peaking in women aged 25‑29 and lowest in those ≥35. Primiparas showed markedly higher PROM rates than multiparas each year (2018: 29.8% vs. 3.9%; 2019: 22.9% vs. 7.3%; 2020: 23.3% vs. 6.2%; all p < 0.0001). PROM was likewise more frequent in pregnancies that ultimately required cesarean section (17.5‑20.2%) than in vaginal births (9.3‑11.1%) during each study year (all p < 0.0001). No significant month‑to‑month or seasonal trend was detected (χ² = 0.50; p = 0.779).

Conclusions: In this temperate‑climate cohort, PROM clustered in younger, first‑pregnancy mothers and was strongly associated with subsequent cesarean delivery, while advanced maternal age and seasonality exerted minimal influence. These findings highlight nulliparous women in the 20-29-year age group as a key population for targeted education, intensified surveillance, and early labor management planning. Future prospective studies should explore the biological mechanisms underlying the age‑parity interaction and evaluate interventions, such as infection screening or cervical support, that may mitigate PROM risk in this high‑incidence group.

## Introduction

Premature rupture of membranes (PROM) is defined as the rupture of fetal membranes before the onset of labor, regardless of gestational age. PROM can be further classified into term PROM, which occurs at or after 37 weeks of gestation, and preterm PROM (PPROM), which refers specifically to membrane rupture before 37 weeks of gestation. It is a common obstetric complication, affecting approximately 8-10% of all pregnancies [1,2]. In addition to neonatal complications (such as respiratory distress, sepsis, and cord prolapse) and maternal risks (chorioamnionitis and endometritis) [3], PROM poses management dilemmas regarding the timing of delivery and infection prevention [4]. The clinician caring for a pregnant woman with PROM is central to effective management and should possess a thorough understanding of potential complications and appropriate interventions to minimize risks and optimize outcomes [5]. Understanding PROM's epidemiology and risk factors is highly important given these implications.

In prior studies, maternal demographic factors such as age and parity have shown inconsistent associations with PROM [3,4,6,7]. Some reports suggest that extremes of maternal age heighten the risk: mothers under 20 years may have a higher PROM incidence (possibly related to cervical immaturity), and advanced maternal age (commonly defined as >35 years) has also been cited as a risk factor in specific populations [3]. Parity has likewise been debated; while uterine overdistension in grand multiparas (due to multiple gestation or polyhydramnios) can precipitate membrane rupture, other studies have found nulliparity to be a risk factor, noting that first pregnancies may lack the cervical remodeling that occurs in later pregnancies [3,6]. Additionally, there is a paucity of data on how the mode of delivery correlates with PROM risk, aside from the known observation that PROM can lead to an increased likelihood of operative delivery [4]. Seasonal variation in PROM rates is another understudied area; some have hypothesized that climate factors (temperature, humidity) or seasonal infection patterns could influence PROM, but evidence is limited and mixed [7].

Given these gaps in knowledge, we conducted a three-year retrospective analysis focusing on maternal age, parity, delivery mode, and seasonality as determinants of PROM. We aimed to clarify how these factors relate to our population's PROM incidence and compare our findings with established literature. By better delineating these associations, this study seeks to improve risk stratification for PROM and inform clinical decision-making for at-risk pregnancies.

## Materials and methods

Study design and population

This retrospective study included parturients who delivered at Dongguan Eighth People's Hospital, Dongguan, China, between January 1, 2018, and December 31, 2020. A total of 13,209 deliveries were recorded, and 1,761 cases were identified as PROM. Informed consents were obtained from the patients. The authors affirm that all procedures contributing to this work comply with the ethical standards of the relevant national and institutional committees on human experimentation and the Helsinki Declaration of 1975, as revised in 2013.

Inclusion and exclusion criteria

The inclusion criteria for PROM encompass the following: singleton pregnancy with cephalic presentation; confirmed membrane rupture through biochemical tests (ferning/nitrazine); gestational age ranging from 16+0 to 41 completed weeks, which was established using either ultrasound dating or the date of the last menstrual period (LMP); and admission within six hours of rupture with cervical dilation <3 cm.

The exclusion criteria for PROM include the following: signs of chorioamnionitis (such as fever, uterine tenderness, foul-smelling amniotic fluid, or fetal tachycardia); the presence of meconium-stained amniotic fluid; active labor or regular uterine contractions; significant fetal anomalies or chromosomal abnormalities; multiple pregnancies; maternal medical or obstetric complications requiring immediate delivery (e.g., preeclampsia); intrauterine fetal demise prior to rupture; cervical cerclage in place; previous chorionic villus sampling (CVS) in the current pregnancy; uterine structural anomalies; and large uterine fibroids (>6 cm).

Data collection

Data were extracted from hospital records, including the month of delivery, maternal age, parity (primiparous or multiparous), and mode of delivery (vaginal or cesarean). PROM cases were defined based on clinical diagnoses and confirmed using amniotic fluid leakage tests.

## Results

Comparison of PROM incidence among maternal age groups

**Figure 1:**
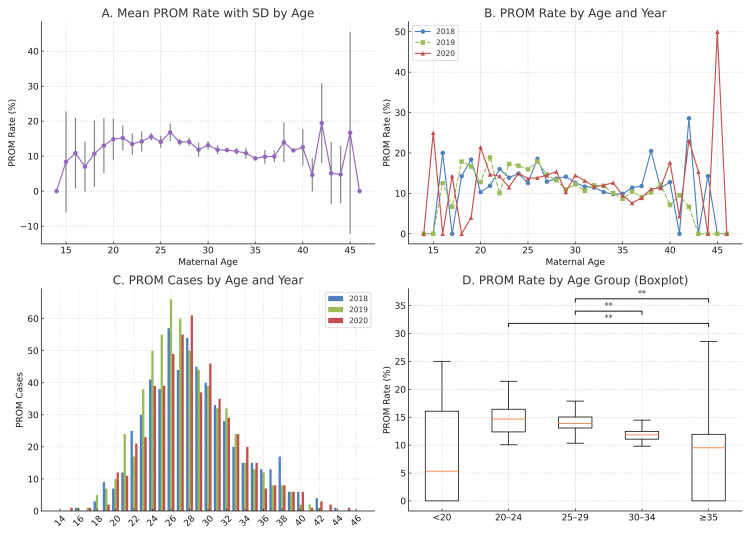
Comparison of PROM incidence among maternal age groups. (A) Average PROM incidence (%) with SD by maternal age, calculated from combined 2018-2020 data. (B) Annual PROM incidence (%) stratified by maternal age for each year (2018, 2019, 2020). (C) Year-specific PROM case counts by age in a grouped bar chart format. (D) Distribution of PROM rates by maternal age group: <20, 20-24, 25-29, 30-34, and ≥35 years. Statistical differences were tested using the Mann-Whitney U test with Holm-Bonferroni correction. **p < 0.01. PROM: premature rupture of membranes; SD: standard deviation

Maternal age was analyzed in five aggregated groups to avoid instability due to sparse data in certain ages: <20, 20-24, 25-29, 30-34, and ≥35 years. Figure 1A displays PROM incidence by individual age, with visible fluctuation at less-represented ages. Figure 1B shows year-specific trends that were broadly similar from 2018 to 2020. Most PROM cases occurred in women aged 24-30 (Figure 1C), consistent with the overall distribution of deliveries. Statistical comparison among age groups (Figure 1D) using the Kruskal-Wallis test demonstrated a significant difference (H = 19.95; p = 0.00051). Post hoc Mann-Whitney U tests with Holm-Bonferroni correction revealed significantly higher PROM rates in the 25-29 group (14.15 ± 2.21) compared to 30-34 (11.77 ± 1.24; p = 0.0012), in the 20-24 group (14.65 ± 3.16) compared to ≥35 (9.82 ± 9.88; p = 0.0010), and in the 20-24 group compared to 30-34 (p = 0.0027).

Comparison of PROM incidence between primiparas and multiparas

**Figure 2:**
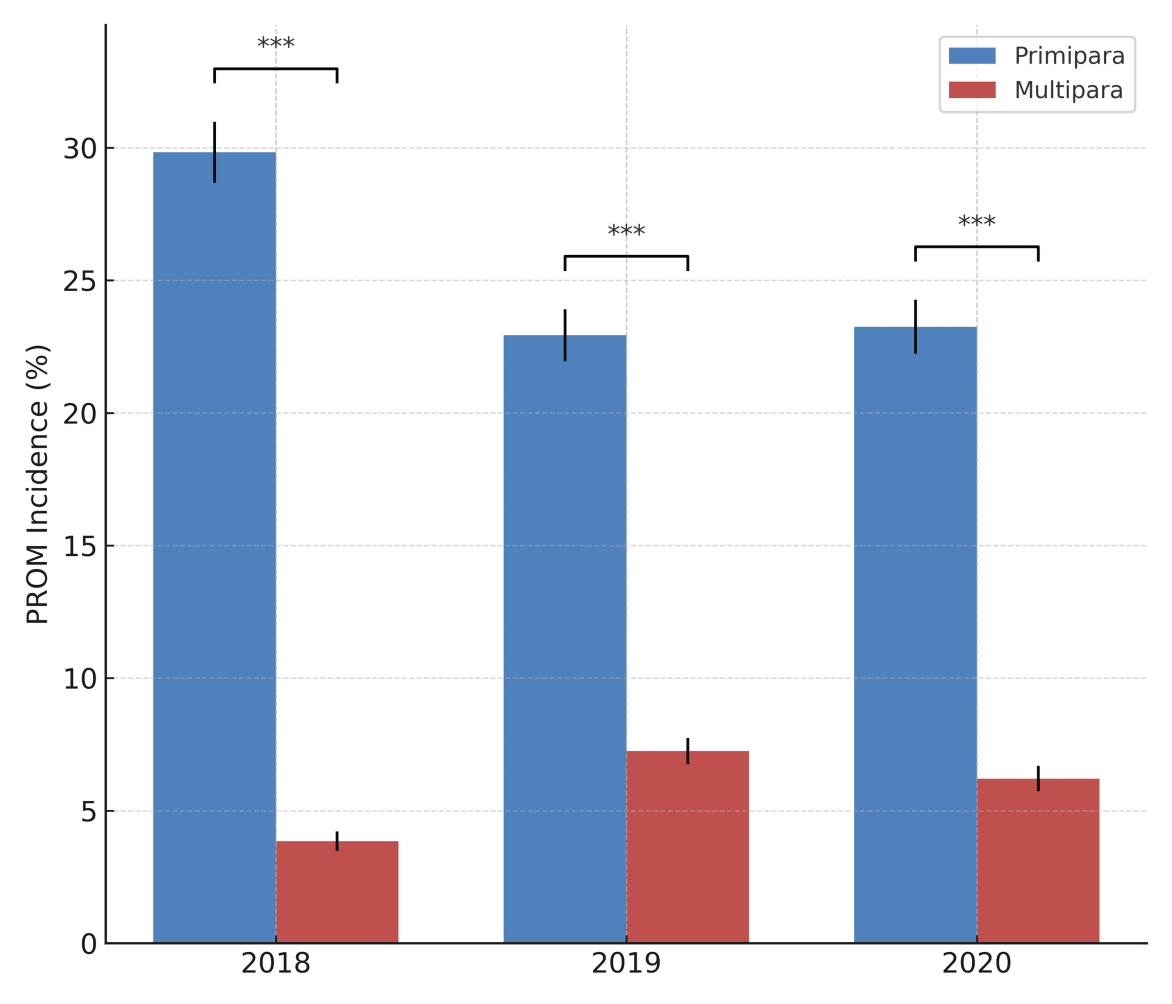
Comparison of PROM incidence between primiparas and multiparas. Bar chart comparing the incidence of PROM between primiparas and multiparas over three consecutive years. Error bars represent standard error of proportions. PROM incidence was consistently and significantly higher in primiparas compared to multiparas across all years (chi-squared test: χ² = 584.33 and p < 0.0001 for 2018; χ² = 230.15 and p < 0.0001 for 2019; χ² = 261.62 and p < 0.0001 for 2020). ***p < 0.001 PROM: premature rupture of membranes

To assess the impact of parity on PROM incidence, delivery data were stratified by parity from 2018 to 2020. Each year, PROM incidence was significantly higher in primiparas than in multiparas: 29.82% vs. 3.85% in 2018, 22.93% vs. 7.25% in 2019, and 23.25% vs. 6.21% in 2020. Chi-squared tests confirmed that these differences were highly significant in all three years (p < 0.0001). Figure 2 visually illustrates the consistent disparity between the two groups.

Comparison of PROM incidence between vaginal and cesarean deliveries

**Figure 3:**
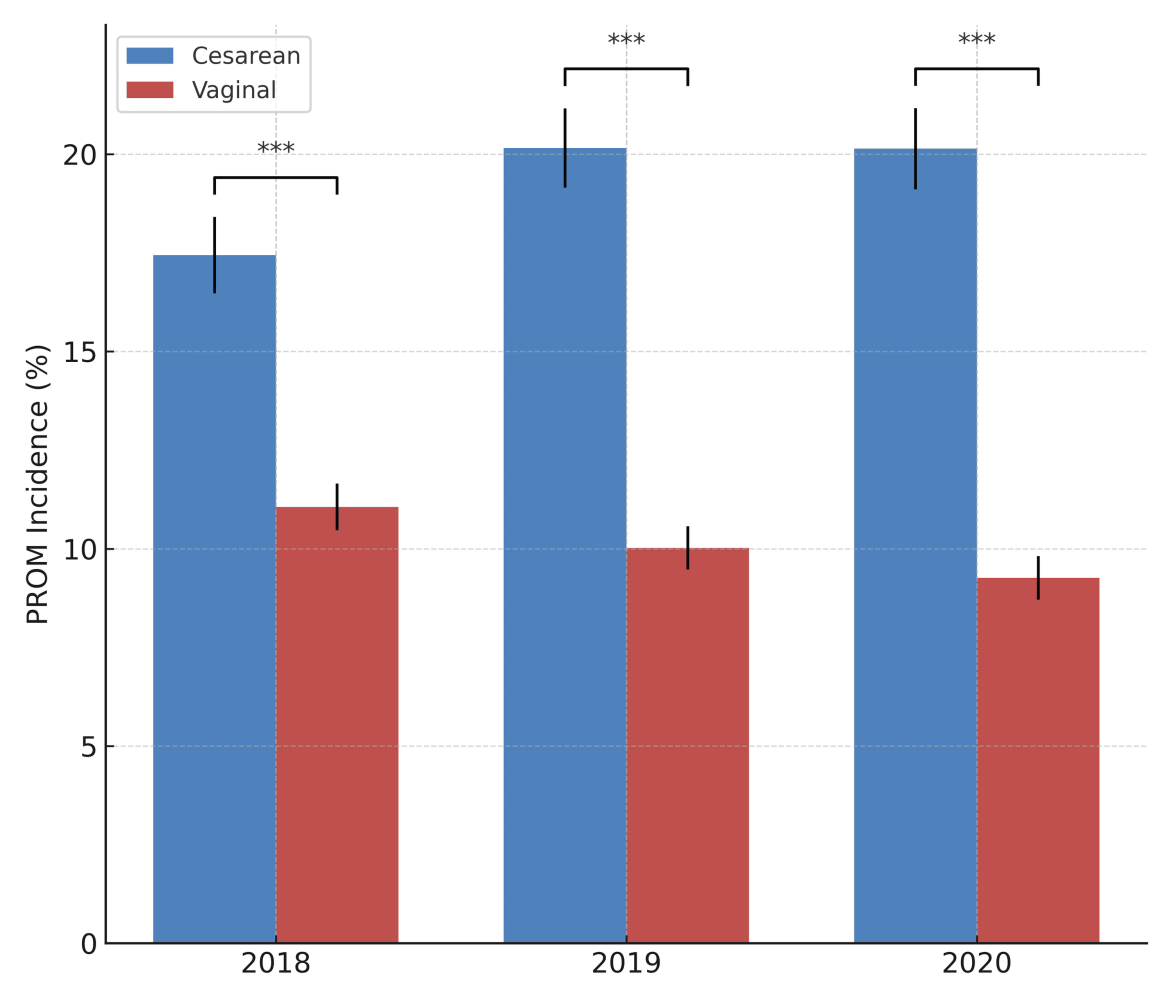
Comparison of PROM incidence between vaginal and cesarean deliveries. Bar chart comparing the incidence of PROM in women undergoing cesarean section and vaginal delivery across three consecutive years. Error bars represent the standard error of proportions. PROM incidence was significantly higher in cesarean deliveries compared to vaginal deliveries in each year (chi-squared test: χ² = 34.43 and p < 0.0001 for 2018; χ² = 90.52 and p < 0.0001 for 2019; χ² = 101.01 and p < 0.0001 for 2020). ***p < 0.001 PROM: premature rupture of membranes

The incidence of PROM was compared between cesarean and vaginal delivery groups over a three-year period. In 2018, PROM occurred in 17.45% of cesarean deliveries and 11.06% of vaginal deliveries. In 2019, the corresponding rates were 20.16% vs. 10.02% and, in 2020, 20.14% vs. 9.25%. In all three years, PROM incidence was significantly higher in the cesarean group compared to the vaginal group. Chi-squared tests confirmed highly significant differences yearly (p < 0.0001), as visually summarized in Figure 3.

No significant seasonal variation in PROM incidence across 2018-2020

**Figure 4:**
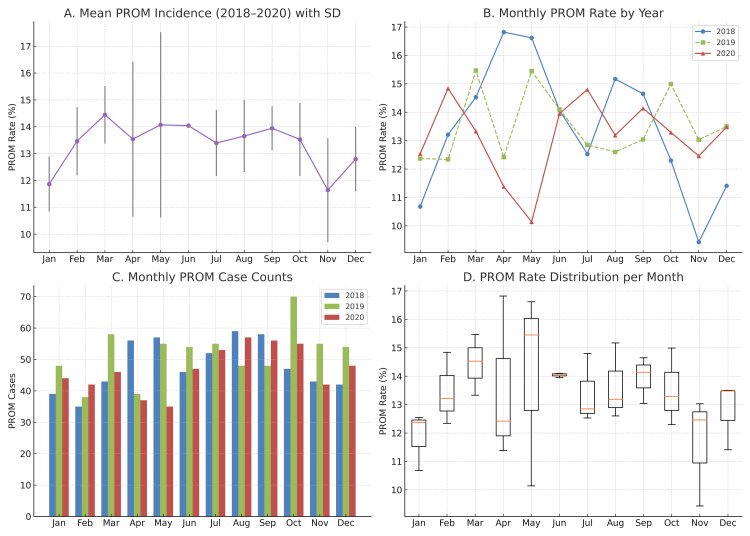
No significant seasonal variation in PROM incidence across 2018-2020. (A) Mean monthly PROM incidence across 2018-2020 with SD error bars. (B) Monthly PROM incidence (%) by year. (C) Monthly PROM case counts for each year. (D) Distribution of PROM rates by month across the three years shown as boxplots. No statistically significant differences in PROM incidence were observed between months (Friedman test: χ² = 0.50; p = 0.779). PROM: premature rupture of membranes; SD: standard deviation

The monthly incidence of PROM was analyzed over a three-year period. The mean PROM rate for each calendar month is presented in Figure 4A, with error bars representing the standard deviation from 2018 to 2020. PROM incidence ranged from 11.61% in January to 16.62% in May, showing minor fluctuations throughout the year. The monthly PROM rates for each year demonstrated similar seasonal profiles, with no consistently recurring peak months across the three years (Figure 4B). PROM case counts were also relatively evenly distributed throughout the year (Figure 4C). Boxplot analysis comparing monthly PROM rates across 2018, 2019, and 2020 (Figure 4D) did not reveal significant differences. The Friedman test confirmed no statistically significant variation in monthly PROM incidence (χ² = 0.50; p = 0.779), suggesting a stable month-to-month pattern across the study period.

## Discussion

We observed notable relationships between PROM incidence and maternal characteristics in this retrospective cohort. Maternal age had a significant but non-linear association with PROM. Women in the 20-29-year age group experienced the highest PROM rates, whereas those of advanced age (≥30, especially ≥35) had a lower incidence of PROM. This finding contrasts somewhat with the traditional expectation that very young or older mothers are at greater risk [3]. Prior studies indeed have reported that teenage mothers have an elevated risk of PROM, potentially due to biological immaturity or socioeconomic factors [3]. Likewise, advanced maternal age has been linked to membrane fragility and increased PROM in some analyses. For example, one recent cohort from China noted that women aged 35-39 had significantly higher odds of PROM than those in the 20-29-year age group [6]. However, our data did not show an increased PROM rate in the older age groups; the ≥35-year group had one of the lowest incidences. Our findings underscore that the influence of maternal age on PROM is complex. It may be population-specific and confounded by parity and clinical management factors. Overall, the result that the 20-29-year age group was most affected suggests that age alone is not a simple linear predictor of PROM risk, aligning with the multifactorial nature of this condition [8].

In our analysis, parity demonstrated a robust and transparent relationship with PROM. Across all three years, primiparous women had a dramatically higher incidence of PROM than multiparous women (on the order of three- to sevenfold, depending on the year). This is consistent with some prior research. Liao et al. reported that nulliparity was associated with approximately 2.5-fold higher odds of PPROM in a large prospective cohort [6]. Similarly, a community-based study observed that the combination of first pregnancy and advanced maternal age significantly increased PROM risk (OR ~6.8) compared to younger multiparas [6]. The biological reasons for higher PROM frequency in first pregnancies are not fully established. One hypothesis is that cervical connective tissue may be less distensible or resilient before it has undergone the remodeling that occurs in a prior delivery, making it more prone to rupture under stress. Nulliparous women may also have longer labors on average, increasing the duration of membrane exposure to contractions if labor is protracted. On the other hand, multiparous women might have more efficient labor or perhaps stronger amniotic membranes (if they did not experience PROM in earlier pregnancies, it could indicate a lower inherent predisposition). It's worth noting that some earlier studies have suggested high parity can be a risk due to uterine overdistension in grand multiparity, but in our modern obstetric population, grand multiparity is less common, and factors like the history of PROM or cervical procedures might play a more significant role than the absolute parity number [3]. Our findings reinforce that primigravida represents a high-risk group for PROM, which has important clinical implications: primigravids may benefit from closer surveillance for signs of PROM (such as regular checks for fetal fibronectin or cervical length in those with symptoms) and patient education about reporting fluid leakage.

We found an intriguing association between PROM and delivery mode. Specifically, pregnancies that were ultimately delivered via cesarean section had a significantly higher incidence of PROM compared to those delivered vaginally. This may seem counterintuitive, since one might expect that if membranes rupture early, labor often ensues and could lead to vaginal birth. However, there are several plausible explanations supported by prior studies. First, PROM can alter the course of labor and increase the likelihood of cesarean delivery. For instance, if PROM occurs without active labor (especially in a preterm or term but unfavorable cervix scenario), it can prompt early intervention or induction that might culminate in a cesarean if labor does not progress. Consistent with our findings, a 2024 cohort comparing expectant management with elective delivery at 34 weeks reported significantly higher operative delivery rates among women experiencing PPROM [9]. Bond et al. reported in a Cochrane review that among women with PPROM, those managed with immediate delivery (as opposed to expectant management) had a significantly higher rate of cesarean section (RR: 1.26) [4]. This suggests that PROM often leads to more frequent operative delivery, likely due to complications such as malpresentation, fetal distress, or failed induction. In our data, the higher PROM incidence in the cesarean group could be partly because some women had PROM as the event that precipitated the decision for a cesarean (for example, a term PROM in a patient with a prior uterine scar may prompt a repeat cesarean rather than a trial of labor). Additionally, certain risk factors (like prior PROM or uterine scarring) overlap with both higher PROM risk and higher chance of cesarean. Our finding aligns with these considerations. Clinically, it highlights that women who experience PROM should be counseled about an increased possibility of cesarean delivery and obstetric teams should be prepared for operative intervention if indicated. It also raises the point that preventing PROM (when possible) might reduce downstream unplanned cesareans.

Finally, our analysis showed no significant seasonal variation in PROM incidence over the three-year period. The monthly PROM rates remained relatively stable, with only minor fluctuations, and statistical testing confirmed no difference in incidence between summer, winter, or other seasons. This indicates that, in our region (Dongguan, China), seasonality was not a significant factor in PROM occurrence. This finding aligns with some prior observations that spontaneous obstetric events like PROM or preterm labor may not follow a strict seasonal pattern in temperate climates [10]. However, it contrasts with studies that have found environmental conditions can influence PROM when extreme [7,11-13]. For example, Gat et al. noted that high ambient temperatures were an independent risk factor for PPROM among Bedouin-Arab women in the Negev desert. In that population, very hot weather conditions were associated with more frequent membrane rupture, presumably due to dehydration or heat stress affecting membrane integrity [7]. Another recent study by Jiao et al. in Southern California examined heat waves and found that acute extreme heat exposure significantly increased the risk of PROM [11]. Interestingly, the heat-related PROM risk in that study was notably elevated in subgroups of mothers under 25 years old and those of lower socioeconomic status, suggesting that younger, potentially more vulnerable pregnant women are disproportionately affected by environmental heat stress [11]. Given those findings, why did we not observe any seasonal effect? It could be that our local climate variations were not extreme enough during 2018-2020 to impact PROM rates (for instance, if summers were warm but not at a level of heat stress and winters were mild). Additionally, our hospital's patient population might have good access to indoor climate control and healthcare resources year-round, buffering any environmental influences. Our data's lack of a seasonal trend implies that intrinsic factors (like the maternal and pregnancy characteristics discussed above) outweighed any extrinsic seasonal factors in determining PROM risk. 

This retrospective audit has several important constraints. First, key confounders, such as maternal body mass index, smoking, genitourinary infection, and history of cervical procedures, were not captured, so residual confounding cannot be excluded. Second, all deliveries came from a single urban tertiary center, limiting generalizability to other practice settings. Third, PROM cases were not stratified into preterm versus term subgroups; therefore, age- and parity-specific patterns observed here may differ between these clinically distinct entities. Confirmation in larger, prospectively collected multicenter cohorts is needed.

Despite these constraints, the demographic signals detected, especially the pronounced susceptibility of primigravid women in their mid-20s, offer pragmatic levers for risk-stratified care. Electronic health record dashboards could automatically flag nulliparous patients aged 20-29 years for the following: (1) targeted counselling on early signs of membrane rupture and the importance of prompt presentation; (2) low-threshold outpatient screening (e.g., repeat vaginal pool pH testing or fetal fibronectin assays when symptoms arise); and (3) algorithm-driven obstetric triage that incorporates age, parity, and prior cervical procedures to prioritize timely review in units with limited capacity.

## Conclusions

In a temperate, single‑center population, PROM clustered in younger primiparas and in pregnancies delivered by cesarean, while advanced maternal age and seasonality played minimal roles. These results advocate heightened surveillance for first pregnancies and early counselling about delivery contingencies when PROM occurs and suggest that preventive strategies should target demographic rather than climatic risk factors.
